# Gossip in the Dictator and Ultimatum Games: Its Immediate and Downstream Consequences for Cooperation

**DOI:** 10.3389/fpsyg.2019.00651

**Published:** 2019-03-28

**Authors:** Junhui Wu, Daniel Balliet, Yu Kou, Paul A. M. Van Lange

**Affiliations:** ^1^ Beijing Key Laboratory of Applied Experimental Psychology, National Demonstration Center for Experimental Psychology Education, Institute of Developmental Psychology, Beijing Normal University, Beijing, China; ^2^ Department of Experimental and Applied Psychology, Vrije Universiteit Amsterdam, Amsterdam, Netherlands

**Keywords:** gossip, cooperation, dictator game, ultimatum game, trust, reciprocity

## Abstract

In this research, we examine how cooperation emerges and develops in sequential dyadic interactions when the initial interaction varies in strategic considerations (i.e., fear of partner rejection) or potential gossip by one’s partner that may affect subsequent interactions. In a lab experiment involving real-time interactions (*N* = 240) across 39 sessions, participants acted in different roles (i.e., Person A, B, and C) in two different games—Person A was first assigned to allocate an amount of resource to Person B in a dictator game or an ultimatum game. Afterward, Person C interacted with Person A (i.e., trustee) as a trustor in a trust game. Prior to their decisions, participants (a) learned that Person B could gossip by sending evaluations about Person A’s behavior to Person C prior to the trust game or (b) did not receive this information. Findings replicate previous research showing that potential gossip by one’s partner greatly increases cooperation that is revealed in the resources allocated to the partner. Yet, compared to the dictator game, the presence of strategic considerations in the ultimatum game does not significantly enhance cooperation, and even makes people less likely to reciprocate others’ behavior in the subsequent interaction. Interestingly, when there is no gossip, those who have played the ultimatum game, compared to the dictator game, are more trusted by others but do not vary in reciprocity in the subsequent interaction. However, when there is gossip, those who have played the dictator game, compared to the ultimatum game, are more trusted and also more likely to reciprocate others’ behavior in the subsequent interaction. These findings imply that gossip invariably promotes cooperation across strategic and non-strategic situations, but the potential rejection by one’s partner weakly promotes cooperation, and even undermines future cooperation especially when paired with reputation sharing opportunities. We discuss the implications of these findings for implementing reputation systems that can promote and maintain cooperation cost-effectively.

## Introduction

Fairness and cooperation are important elements for the effective functioning of groups, organizations, and the society at large. Yet, achieving mutual cooperation is often challenging, because people recurrently face various resource allocation trade-offs between self and others. Such trade-offs make it tempting for individuals to pursue their own personal interest, which may lead to the breakdown of cooperation (Rand and Nowak, 2013). For decades, a large body of literature across disciplines has sought to explain why people cooperate by paying a personal cost to benefit others in various social interaction contexts (for reviews, see Nowak, 2006; Kurzban et al., 2015; Wu et al., 2016b). One explanation is that cooperation increases when people repeatedly interact with others over an extended period of time (Trivers, 1971; Van Lange et al., 2011). Yet, social interactions very often involve one-shot interactions with strangers who are not able to reciprocate later. Why do people still cooperate for the welfare of unknown others in such situations?

Models of indirect reciprocity may account for the prevalence of cooperation in one-shot interactions with others (Nowak and Sigmund, 2005). Indirect reciprocity describes the phenomenon that one’s current cooperative behavior is reciprocated by other third parties in future interactions, and this process is facilitated by the transmission of one’s reputation through either direct experience or gossip among interaction partners across different situations (e.g., Bromley, 1993; Anderson and Shirako, 2008). Having a good reputation brings about potential indirect benefits in various forms (e.g., resources, attraction to coalition partners), whereas a bad reputation may relate to indirect costs in terms of social exclusion or third-party punishment. For example, behavioral experiments using donation games suggest that donors tend to donate more frequently to recipients who have been generous to others in previous interactions (Wedekind and Milinski, 2000; Wedekind and Braithwaite, 2002). Some recent field research has also documented such tendencies to favorably treat others with a good reputation, such as providing free services to requesters who have helped others on online service platforms (van Apeldoorn and Schram, 2016), offering more tips to hairdressers who have engaged in charity fundraising (Khadjavi, 2016), and trusting Airbnb users with more positive ratings and reviews (Abrahao et al., 2017). In addition, explicit or implicit reputational cues in the situation can also enhance cooperation. For example, in public, compared to anonymous, situations people are more likely to contribute to public goods, behave prosocially, and punish norm violators at a personal cost (Kurzban et al., 2007; Simpson and Willer, 2008; Van Vugt and Hardy, 2010). In addition, gossip enables reputation to circulate and spread in larger social networks (Dunbar, 2004), and can facilitate partner selection and cooperation in a cost-effective way (Beersma and Van Kleef, 2011; Feinberg et al., 2012; Giardini and Conte, 2012). Indeed, both the threat of potential gossip and the actual reputation spreading through gossip have been found to positively enhance cooperation in social interactions (Piazza and Bering, 2008; Beersma and Van Kleef, 2011; Feinberg et al., 2014). Notably, some recent evidence even suggests that gossip or a desire to establish a positive reputation can promote and maintain cooperation more effectively and efficiently than punishment (Grimalda et al., 2016; Wu et al., 2016a). Here, we seek to replicate the positive effect of gossip on cooperation in dyadic interactions. Our prediction is that potential gossip by one’s partner would make people more cooperative toward others (*Hypothesis 1*).

Notably, such reputation-based cooperation may also depend on the specific interaction context. Here, we focus on social interactions that involve strategic considerations. The difference in strategic considerations can be illustrated in two economic games involving dyads—the dictator game (DG) and the ultimatum game (UG). In a standard dictator game, participants acting as the allocator can freely divide an amount of resource with a receiver, who has no choice but to accept the offer (Forsythe et al., 1994). Thus, the DG allocators’ behaviors reveal their pure preferences for fairness and concerns for others’ welfare. Different from the dictator game, in a standard ultimatum game, the proposer makes an offer regarding how to split an amount of resource with the responder, who can accept or reject this offer. If this offer is accepted, then the resource is split as proposed. If this offer is rejected, then both earn nothing (Sanfey et al., 2003; Güth and Kocher, 2014). Thus, the UG proposers’ behaviors may be motivated by fear of rejection of unfair offers, apart from the preference for fairness that exists in the dictator game (Van Dijk and Vermunt, 2000). The strategic considerations due to fear of rejection in the ultimatum game may elicit more cooperation than the dictator game. Indeed, previous research has consistently found higher proportion of resources offered to the partner in the ultimatum game, compared to the dictator game (Steinbeis et al., 2012; Bechler et al., 2015; Espín et al., 2016). Thus, we predict that people would be more cooperative in an ultimatum game than in a dictator game (*Hypothesis 2a*).

Yet, such strategic interactions in the ultimatum game may make people less intrinsically motivated to cooperate in future interactions with others. That is, although people may cooperate due to an extrinsic incentive to avoid potential rejections by their partners in the ultimatum game, this extrinsic incentive may undermine their intrinsic motivation to cooperate and lead to less cooperation in their future interactions with others. In support of this argument, a recent study reveals that people tend to act more selfishly in a dictator game if they have initially played an ultimatum game (Neumann et al., 2018). Similarly, some indirect evidence on punishment suggests that punishment makes people less intrinsically motivated to cooperate, as cooperation significantly decreases in subsequent interactions when punishment is initially implemented but then removed (Fehr and Gächter, 2002; Mulder et al., 2006; Chen et al., 2009; Nelissen and Mulder, 2013). Thus, we predict that people who have initially played an ultimatum game, compared to a dictator game, would be less cooperative in subsequent interactions with others (*Hypothesis 2b*).

If the strategic motivation evoked by the ultimatum game undermines subsequent cooperation due to a decrease in intrinsic motivation, does this negative effect depend on whether there is reputation transmission in the two sequential interactions? Since cooperators who have a good reputation are more likely to receive long-term indirect benefits from other third parties (Wedekind and Braithwaite, 2002; Nowak and Sigmund, 2005), reputation systems (e.g., gossip and reputation sharing) that are implemented in strategic situations (e.g., the ultimatum game) may have downstream consequences for future cooperation. That is, those who are strategically more cooperative to avoid others’ rejection in the ultimatum game (vs. the dictator game) may also utilize the good reputation they have obtained to pursue their long-term self-interest (e.g., mislead others to cooperate with them and exploit these others’ cooperation). Thus, we predict that the initial interaction experience (i.e., a dictator game or an ultimatum game) would interact with the possibility of gossip in predicting subsequent cooperation with others. Specifically, we expect that people who have initially played an ultimatum game, compared to a dictator game, would be less cooperative in subsequent interactions especially when there is gossip transmitted between the two interactions (*Hypothesis 3*).

Taken together, the present research sought to examine how cooperation emerges and develops in sequential dyadic interactions when the initial interaction varies in (a) strategic considerations (i.e., fear of partner rejection) or (b) potential gossip by one’s partner that may affect subsequent interactions. To test our hypotheses about the immediate and downstream effects of gossip and variation in the initial game, we conducted a multi-session lab experiment involving real-time interactions. In each session, groups of participants interacted with others as either Person A, B, or C across two sequential games: Person A first allocated an amount of resource to Person B (i.e., receiver or responder) in a dictator game or an ultimatum game, then interacted with Person C (i.e., trustor) as a trustee in a trust game. In half of the situations, Person B’s evaluations about Person A’s behavior were sent to Person C prior to the trust game (i.e., gossip manipulation; see also Wu et al., 2015). This experimental setting also enabled us to test the open questions about whether the initial game and gossip manipulation affect (a) Person B’s emotional responses and evaluations about Person A’s behavior, and (b) Person C’s trust decisions toward Person A. Participants were provided with performance-based incentives and the study did not use any deception.

## Materials and Methods

### Participants and Design

We recruited 240 students (137 women, *M*_age_ = 21.53 years, *SD* = 3.44) from a Dutch university to participate in this experiment. The experiment was a 2 (initial game: dictator game, ultimatum game) × 2 (gossip: gossip, control) between-participants design with four conditions. All participants provided their written informed consent prior to the experiment. They received a baseline payment of €2 and could earn an extra bonus of up to €4 based on their own and others’ decisions across two sequential games. On average, they earned €1.12 (*SD* = 0.76) as a bonus and thus received an overall payment of €3.12.

### Procedure

We conducted this experiment in a psychology lab with separate cubicles using the Software Platform for Human Interaction Experiments (SoPHIE; Hendriks, 2012). Data were collected across 39 sessions with 6 or 12 participants in each session. We aimed to recruit at least six participants in each session to guarantee that they could not tell who would interact with them during the experiment. Participants in the same session were assigned to the same condition. Assignment to the four conditions was not random but was unrelated to participant characteristics of age and gender (*r*s from −0.08 to 0.18, *p*s > 0.11). Participants were informed to interact with others in two decision-making tasks: (a) a dictator game or an ultimatum game involving Person A and Person B, and (b) a subsequent trust game involving Person C and Person A. They first read about the two games and answered several comprehension check questions. Explanations were provided to them if they answered any question incorrectly. Then they were randomly assigned as Person A, B, or C after they correctly answered all the questions. Thus, the 240 participants were divided into 80 triads including Person A, B, and C, with 20 triads in each condition.

#### Dictator Game (DG) or Ultimatum Game (UG)

The dictator game involved an allocator (Person A) and a receiver (Person B). The allocator could freely allocate 100 monetary units (MUs) between him/herself and the receiver, while the receiver had no choice but to accept the allocated amount (Forsythe et al., 1994). They both learned about their outcomes after the allocator made the decision. The ultimatum game involved a proposer (Person A) and a responder (Person B). The proposer initially received 100 MUs, and could offer any number of MUs to the responder, who could then accept or reject this offer. If this offer was accepted, the 100 MUs would be divided as proposed. If it was rejected, both would earn nothing (Sanfey et al., 2003). They both learned about the proposed offer, whether it was accepted or rejected, and their outcomes after their respective decisions. The number (range: 0–100) of MUs that Person A gave to Person B in the dictator game or the ultimatum game was our measure of generosity.

#### Trust Game (TG)

The trust game involved a trustor (Person C) and a trustee (Person A). Across all conditions, the trustors were not informed about the exact number of MUs that the trustees allocated to their partners in the initial game. The trustor was initially endowed with 100 MUs and could send any number of MUs to the trustee. The amount sent to the trustee was tripled, and the amount that the trustor kept for him/herself retained the same value. After receiving the tripled amount, the trustee chose to send some MUs back to the trustor (Berg et al., 1995). The number of MUs the trustor sent to the trustee and the proportion of MUs returned back by the trustee in this game were the measures of trust and reciprocity, respectively.

#### Gossip Manipulation

**Figure 1:**
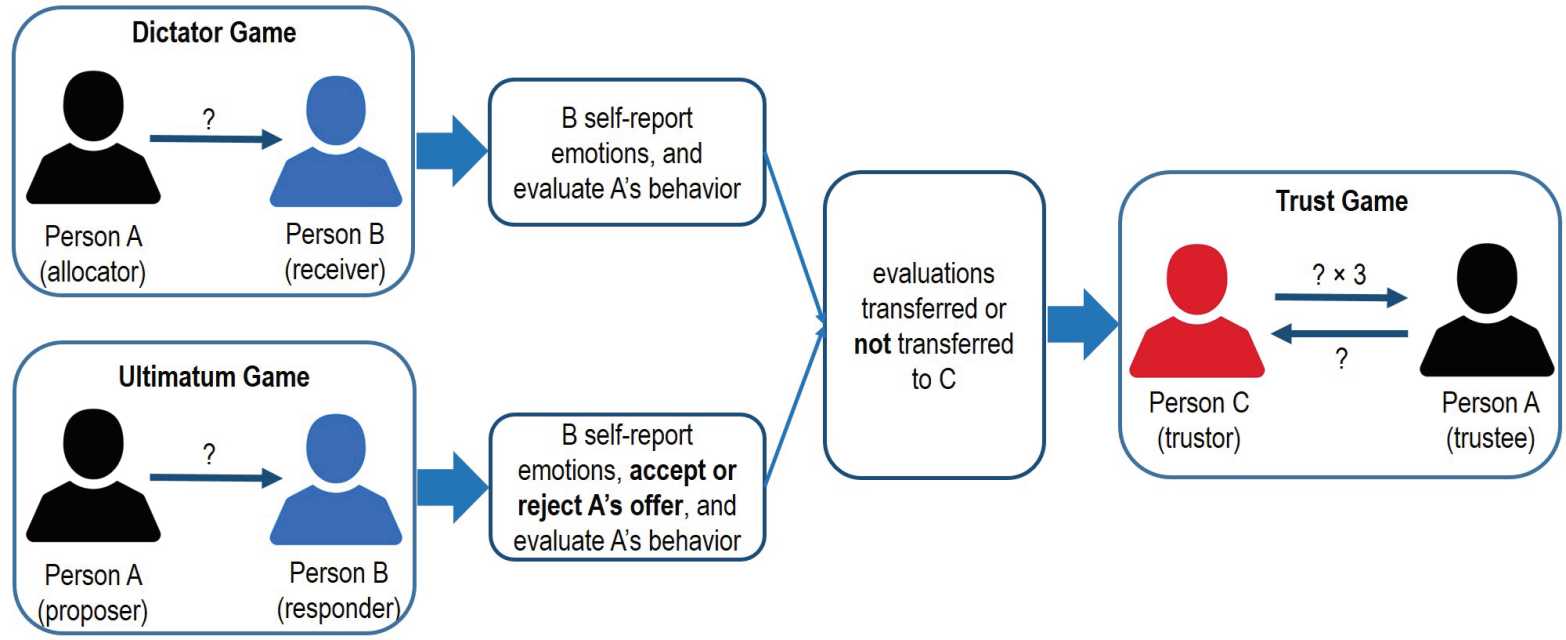
Procedure of the experiment.

After Person A made the decision in the dictator game or the ultimatum game, those acting as Person B learned about Person A’s decision and reported their positive emotions (*α* = 0.88; *elated*, *excited*, *happy*, *relieved*), negative emotions (*α* = 0.58; *angry*, *disappointed*, *frustrated*, *irritated*), and evaluations about Person A’s behavior (*α* = 0.88; *trustworthy*, *friendly*, *considerate*, *generous*) on 7-point Likert scales (1 = *not at all*, 7 = *extremely*). Participants’ average scores across all items of each measure represented their positive and negative emotions, as well as their positive evaluations about Person A. In the ultimatum game, those acting as Person B reported their evaluations after they made their decision to accept or reject the proposer’s offer. Prior to making their decisions in the two tasks, half of the participants learned that Person B’s evaluations about Person A’s behavior in the first game would be displayed on Person C’s computer screen before Person C made the decision in the trust game (*gossip* condition), while the other half did not receive this information (*control* condition). The evaluations about Person A were actually presented or not presented to Person C depending on whether there was gossip (see Figure 1).

Participants reported their age and gender after they made their decisions in the two tasks. We also measured their social value orientation (i.e., number of prosocial choices) using the nine decomposed games of the triple-dominance measure (Van Lange et al., 1997). Social value orientation refers to one’s relatively stable preference in resource allocations between self and others, and predicts decisions in dictator games, ultimatum games, and trust games (Van Lange et al., 1997; Hilbig and Zettler, 2009; Kanagaretnam et al., 2009). Thus, we controlled for its effect when testing our hypotheses.

## Results

### Generosity

**Figure 2:**
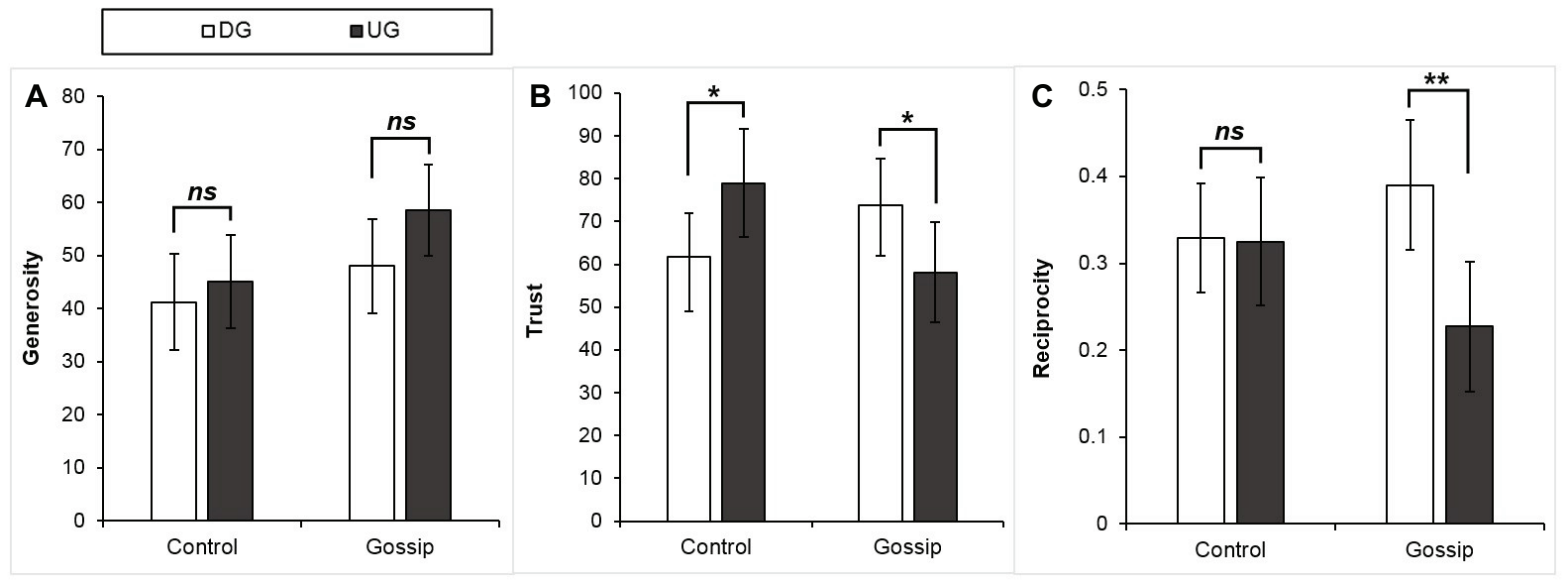
Means levels of **(A)** generosity, **(B)** trust, and **(C)** reciprocity as a function of the initial game and gossip manipulation. Reciprocity was the proportion of MUs from the received tripled amount that Person A sent back to Person C in the trust game. DG, dictator game; UG, ultimatum game. Error bars represent 95% confidence intervals. ^*^ *p* < 0.05, ^**^ *p* < 0.01.

On average, participants assigned as Person A allocated 48.20 MUs (*SD* = 20.75) to Person B. In the ultimatum game, only 1 out of 40 participants who acted as the responder rejected the proposer’s offer. To test whether people are more cooperative when their partner can gossip (vs. cannot gossip; Hypothesis 1) and when they act as UG proposers (vs. DG allocators; Hypothesis 2a), we conducted a 2 (Initial Game) × 2 (Gossip) analysis of covariance (ANCOVA) on generosity with Person A’ social value orientation as the covariate. Participants’ social value orientation was significantly related to their generosity, *F*(1, 75) = 16.78, *p* < 0.001, $\eta_{p}^{2}$ = 0.18. Participants were more generous when their partner could gossip with their future partner (*M* = 53.25, *SD* = 20.49), compared to when their partner could not gossip (*M* = 43.15, *SD* = 20.00), *F*(1, 75) = 4.18, *p* = 0.04, $\eta_{p}^{2}$ = 0.05. The UG proposers (*M* = 51.78, *SD* = 20.92) showed a relatively higher level of generosity than the DG allocators (*M* = 44.63, *SD* = 20.20), but this difference was not statistically significant, *F*(1, 75) = 2.59, *p* = 0.11, $\eta_{p}^{2}$ = 0.03. The Initial Game × Gossip interaction was not significant, *F*(1, 75) = 0.55, *p* = 0.46, $\eta_{p}^{2}$ = 0.007 (see Figure 2A). We found similar effects of the initial game, gossip, and the Initial Game × Gossip interaction (*p*s = 0.12, 0.03, and 0.46) from a two-way analysis of variance (ANOVA) without the covariate.

### Trust

On average, participants acting as the trustor sent 68.16 MUs (*SD* = 26.99) to the trustee in the trust game. To test the extent to which people trust others who have experienced (non-)strategic interactions or potential gossip by others, we conducted a 2 (Initial Game) × 2 (Gossip) ANCOVA on trust with the trustor’s social value orientation as the covariate. Participants’ social value orientation was significantly related to their trust decisions, *F*(1, 75) = 5.81, *p* = 0.02, $\eta_{p}^{2}$ = 0.07. We found no significant effect of the initial game, *F*(1, 75) = 0.001, *p* = 0.98, or gossip, *F*(1, 75) = 0.03, *p* = 0.86. However, the Initial Game × Gossip interaction was significant, *F*(1, 75) = 11.15, *p* = 0.001, $\eta_{p}^{2}$ = 0.13. Further simple effect analysis revealed that when there was no gossip, participants acting as the trustor in the subsequent trust game were more likely to trust the UG proposers (*M* = 79.00, *SD* = 28.41) than the DG allocators (*M* = 61.75, *SD* = 23.75), *F*(1, 75) = 5.85, *p* = 0.02, $\eta_{p}^{2}$ = 0.07, whereas when there was gossip, the DG allocators (*M* = 73.75, *SD* = 24.91) were significantly more trusted than the UG proposers (*M* = 58.15, *SD* = 27.06), *F*(1, 75) = 5.53, *p* = 0.02, $\eta_{p}^{2}$ = 0.07 (see Figure 2B). We found similar effects of the initial game, gossip, and the Initial Game × Gossip interaction (*p*s = 0.89, 0.45, and 0.006) from a two-way ANOVA without the covariate.

### Reciprocity

We used the return ratio (the amount Person A returned divided by the tripled amount received from Person C) as the measure of reciprocity (see also Bellemare and Kröger, 2007).^1^ On average, participants acting as the trustee returned 31.77% (*SD* = 0.17) of the received tripled amount from the trustor. To test whether the UG proposers became less cooperative than the DG allocators in the subsequent interaction (Hypothesis 2b) and the interaction between the initial game and the possibility of gossip in predicting subsequent cooperation (Hypothesis 3), we conducted a 2 (Initial Game) × 2 (Gossip) ANCOVA on reciprocity with the trustee’s social value orientation and earnings in the initial game, and the trustor’s decision as the covariates. The trustee’s social value orientation was significantly related to reciprocal behavior, *F*(1, 72) = 7.43, *p* = 0.008, $\eta_{p}^{2}$ = 0.09, *r* = 0.32, but the trustee’s earnings in the initial game, *F*(1, 72) = 1.31, *p* = 0.26, $\eta_{p}^{2}$ = 0.02, *r* = −0.12, or the trustor’s decision, *F*(1, 72) = 0.03, *p* = 0.86, *r* = 0.10, did not significantly affect reciprocity. There was no significant effect of gossip, *F*(1, 72) = 1.12, *p* = 0.29, $\eta_{p}^{2}$ = 0.02. Overall, the UG proposers (*M* = 0.27, *SD* = 0.17) returned a smaller proportion of MUs in the subsequent trust game than the DG allocators (*M* = 0.36, *SD* = 0.16), *F*(1, 72) = 7.53, *p* = 0.008, $\eta_{p}^{2}$ = 0.10. This main effect of the initial game was qualified by a significant Initial Game × Gossip interaction, *F*(1, 72) = 4.76, *p* = 0.03, $\eta_{p}^{2}$ = 0.06. Further simple effect analysis revealed that when there was no gossip, the DG allocators (*M* = 0.33, *SD* = 0.15) and the UG proposers (*M* = 0.33, *SD* = 0.17) showed no significant difference in reciprocity, *F*(1, 72) = 0.12, *p* = 0.73; when there was gossip, the DG allocators (*M* = 0.39, *SD* = 0.17) returned a larger proportion of MUs to the trustor in the trust game than the UG proposers (*M* = 0.23, *SD* = 0.17), *F*(1, 72) = 12.19, *p* = 0.001, $\eta_{p}^{2}$ = 0.14 (see Figure 2C). We found similar effects of the initial game, gossip, and the Initial Game × Gossip interaction (*p*s = 0.03, 0.61, and 0.04) from a two-way ANOVA without the covariates.

### Auxiliary Analyses

To test whether the allocation behaviors in the DG and UG predict the partners’ emotions and evaluations, we first conducted correlational analyses in the two games (see Table 1). These analyses revealed that (a) higher levels of generosity from the DG allocators were associated with more positive emotions, *r*(38) = 0.55, *p* < 0.001, and less negative emotions, *r*(38) = −0.45, *p* = 0.004, experienced by their partners, (b) the UG proposers’ generosity was not significantly associated with their partners’ positive or negative emotions (*p*s > 0.18), and (c) generosity was more strongly correlated with positive evaluations in the dictator game (*r* = 0.66, *p* < 0.001) than in the ultimatum game (*r* = 0.42, *p* = 0.007). We further conducted regression analyses on participants’ emotions and evaluations, including generosity (centered), the initial game (0 = DG, 1 = UG), and their interaction term as predictors. The Initial Game × Generosity interaction significantly predicted negative emotions, *b* = 0.04, *t*(76) = 2.58, *p* = 0.012, and positive evaluations, *b* = −0.02, *t*(76) = −2.12, *p* = 0.04, but did not significantly predict positive emotions, *b* = −0.02, *t*(76) = −1.65, *p* = 0.10. Further simple slope analyses revealed that (a) generosity negatively predicted negative emotions in the DG, *b* = −0.03, *t*(76) = −3.07, *p* = 0.003, but not in the UG, *b* = 0.005, *t*(76) = 0.54, *p* = 0.59, and that (b) generosity more strongly predicted positive evaluations in the DG, *b* = 0.04, *t*(76) = 5.61, *p* < 0.001, compared to the UG, *b* = 0.02, *t*(76) = 2.75, *p* = 0.007.

**Table 1:** Descriptive statistics and bivariate correlations between generosity and partner’s emotions and evaluations.

	*M* (*SD*)	1	2	3	4
1. Generosity	48.20 (20.75)	—	0.55^***^	−0.45^**^	0.66^***^
2. Positive emotion	3.73 (0.93)	0.21	—	−0.57^***^	0.58^***^
3. Negative emotion	2.38 (1.43)	0.09	−0.11	—	−0.50^**^
4. Positive evaluation	4.62 (1.19)	0.42^**^	0.51^**^	−0.07	—

Correlations in the dictator game (*n* = 40) and the ultimatum game (*n* = 40) are presented above and below the diagonal.

^**^ *p* < 0.01;

^***^ *p* < 0.001.

Notably, the positive evaluations about Person A did not significantly correlate with Person C’s trust decisions when these evaluations were sent to those acting as Person C prior to their decision, *r*(38) = 0.05, *p* = 0.77. This finding suggested that participants did not use others’ evaluations (i.e., gossip) to condition their behavior toward the trustees in the trust game.

## Discussion

Gossip and reputation sharing have been documented as important pathways that facilitate the emergence of cooperation (e.g., Piazza and Bering, 2008; Wu et al., 2015, 2016b). Yet, little is known about whether this positive effect also occurs in social interactions that elicit a strategic motivation (e.g., fear of partner rejection), and how such motivation would affect one’s subsequent interactions with others. To address this question, we conducted a real-time experiment examining the dynamics of cooperation in two sequential dyadic interactions. Specifically, we were interested in examining how people would behave in response to potential gossip by their partner in a dictator game or an ultimatum game, and whether this initial exposure to different situations affects their subsequent behaviors in a trust game with a different partner. In addition, by assigning different roles to participants, we could observe how they respond to others’ behaviors and behave toward others who had experienced situations that varied in strategic motivations or potential gossip by their partners.

Our first hypothesis was about the positive effect of gossip on cooperation. Supporting this hypothesis, we found that gossip enhanced cooperation in both the dictator game and the ultimatum game. Similarly, previous online and lab experiments on group interactions revealed that when group members could gossip about each other, people made more contributions to the public good in multiple rounds of public goods games with different partners (Feinberg et al., 2014; Wu et al., 2016a). This phenomenon of reputation-based cooperation has also been demonstrated in a field experiment in a foraging society (Grimalda et al., 2016). Thus, our own and previous findings consistently suggest that gossip and reputation sharing are important means to facilitate cooperation.

Our second hypothesis related to how strategic considerations (e.g., fear of partner rejection) would affect one’s initial and subsequent cooperation. We expected more cooperation in the ultimatum game than in the dictator game (Hypothesis 2a), but that the UG proposers would display less cooperation than the DG allocators in subsequent interactions with others (Hypothesis 2b). Despite that the UG proposers allocated slightly more resources to their partner than did the DG allocators, this difference was not found to be statistically significant (which was probably due to low statistical power), and thus did not provide support for Hypothesis 2a. Similar to this finding, some evidence reveals a weak or no effect of peer punishment on cooperation (e.g., Wu et al., 2009, 2016a), despite an overall robust effect of punishment on cooperation (for a meta-analysis, see Balliet et al., 2011). Indeed, the similar amount of allocations in the DG and UG with gossip option was easy to interpret, as the DG allocators may be motivated to enhance their cooperation for a good reputation. Yet, the non-significant difference in allocations between the two games in the control condition was inconsistent with some previous evidence (e.g., Bechler et al., 2015), and needs further interpretation. In particular, the overall level of generosity (41.25% of endowment given) in the DG with no gossip was higher than previously reported 28.35% of endowment given in a one-shot DG (Engel, 2011). It is possible that awaiting a future interaction with a new partner in the trust game may trigger strategic behaviors that may or may not come out of a concern for reputation. Future research can address this plausible explanation by examining an extra condition in which no subsequent game is anticipated. Notably, in support of Hypothesis 2b, we found that people were less likely to reciprocate others in the trust game after they had played an ultimatum game, compared to a dictator game. This finding is consistent with recent evidence that people act more selfishly in a dictator game after interacting in an ultimatum game (Neumann et al., 2018). Thus, eliciting the fear of partner rejection may not be a robust means to promote cooperation and may even make people become less intrinsic cooperators in subsequent interactions with others.

Interestingly, we found that more allocated resources related to more positive emotions and less negative emotions experienced by the DG receivers. Yet, the UG offers did not relate to the responders’ emotions, which were in contrast to previous evidence that fair offers elicit positive emotions whereas unfair offers elicit negative emotions in the ultimatum game (e.g., Civai et al., 2010). Moreover, the allocation behaviors in the DG, as compared to those in the UG, were more positively related to partners’ positive evaluations. These divergent findings in the two games are interesting, as they suggest that people may infer about their partner’s genuine concern for others’ welfare based on the specific interaction context. In particular, allocation behaviors in the dictator game may reflect this genuine concern more accurately (Van Dijk and Vermunt, 2000). Yet, when there was gossip transmitted across the two sequential games, the positive evaluations of Person A in the initial game did not relate to Person C’s trust decisions. It is plausible that sharing positive evaluations about Person A can be a form of “direct reciprocity” in response to this person’s generous behavior. Thus, Person A may take this chance to gain a good reputation that does not necessarily reflect their genuine concern for others. In addition, in our experimental settings, the trustor (i.e., Person C) also knew about the situations that other participants (i.e., Person A and B) were facing in the two tasks. Thus, the non-significant correlation between the evaluations of Person A and trust decisions toward this person does not necessarily contradict the commonly observed phenomenon of reputation-based indirect reciprocity (van Apeldoorn and Schram, 2016; Abrahao et al., 2017). Of course, given our low sample size on trust decisions in the condition with gossip option (*n* = 40), we need to be cautious when interpreting this result. To address whether the trustors condition their decisions on the target’s reputation, future studies need to improve the design by (a) comparing gossip from the recipient (i.e., Person B) in the first game with gossip from an irrelevant third party, or (b) comparing the current gossip condition with another condition with information asymmetry (e.g., Person B and C know about the gossip transmission, whereas Person A does not know about the occurrence of gossip). In addition, future research can use larger samples to disentangle how people condition their trust decisions on evaluations about others who have played (a) a dictator game that affords a genuine concern for fairness or (b) an ultimatum game that evokes a strategic motivation (i.e., fear of rejection).

Our final hypothesis related to whether people who have played an ultimatum game would be more likely to utilize the good reputation they have obtained to pursue their long-term personal benefits if reputation sharing occurs. Specifically, we expected that people who have initially played an ultimatum game, compared to a dictator game, would be less cooperative in subsequent interactions especially when there is gossip transmission between the two interactions. Indeed, we found a significant interaction between the initial game and gossip manipulation on reciprocity in the trust game: compared to the DG allocators, the UG proposers were equally likely to reciprocate others when there was no gossip, but were less likely to reciprocate others when there was gossip. Notably, in the no-gossip situation, although the UG proposers were more trusted than the DG allocators (*p* = 0.018), they did not return a larger proportion of MUs than the DG allocators (*p* = 0.82), implying a lower tendency of the UG proposers to reciprocate others than DG allocators. Thus, these findings provided support for Hypothesis 3. Interestingly, we also found a significant interaction effect on trust decisions: people were more likely to trust the UG proposers than the DG allocators when there was no gossip, but were less likely to trust the UG proposers than the DG allocators when there was gossip. These findings suggest that people may hold some lay theories about others’ trustworthiness based on others’ previous social interaction experience. In particular, when there are no other cues available, they may infer that those who have faced potential partner rejection in an ultimatum game may have learned the cooperative norm and would cooperate in the future (although this was actually not the case as revealed in their reciprocal behavior), and thus condition their trust on this inference. Yet, when gossip occurs between the two interactions, such gossip option may be considered as strategic means that UG proposers utilize to pursue their long-term personal interest, and make people doubt the UG proposers’ authentic concern for others even if they receive the reputation score about the UG proposers. Of course, these are only our speculations that need to be tested in future research. Overall, our findings suggest that when there is gossip between sequential interactions, those who have played an ultimatum game (vs. a dictator game) are (a) less likely to be trusted, and (b) less likely to reciprocate others in the subsequent interaction. Importantly, the trustor’s decision was not statistically related to the trustee’s reciprocity, and the correlation between trust and reciprocity was non-significant across all conditions (*r*s from −0.15 to 0.13, *p*s > 0.36), suggesting that the observed difference in reciprocity occurs above and beyond the potential effect of trust decisions. Thus, the potential gossip by one’s interaction partner may lead to a decline in subsequent cooperation when the initial interaction evokes strategic motivations to avoid being rejected.

Taken together, our findings suggest that gossip and reputation sharing can promote cooperation in situations that elicit a pure concern for fairness and others’ welfare (e.g., dictator game) and situations that evoke strategic motivations to avoid rejections by others (e.g., ultimatum game). Yet, the strategic motivation to avoid others’ rejection may undermine one’s future cooperation with different partners, particularly when one is aware that gossip and reputation sharing occur between the initial and subsequent interactions. These findings provide novel and practical insights into how to solve the widespread cooperation problems in organizations and societies. In particular, it emphasizes the utility of gossip and reputation sharing in promoting cooperation in dyadic interactions, but also suggests that one should be cautious about the potential long-term downstream consequences of reputation systems when they are implemented in situations that can evoke a strategic motivation.

## Strengths, Limitations, and Future Directions

The present research harnessed the strength of real-time interactions with no deception to investigate the behavioral dynamics in two sequential interactions. To ensure that participants did not recognize whom they were interacting with, we recruited at least six participants to take part in each session. This experimental setup captures many real-life situations that people often encounter—sequential one-shot interactions with different partners—and thus helps us gain insights into how people make decisions in everyday social interactions. Moreover, the assignment of different roles goes beyond previous research as it enables us to address not only how people behave in response to cues of gossip in strategic and non-strategic situations (e.g., Beersma and Van Kleef, 2011; Wu et al., 2015), but also how their partners evaluate their behavior, as well as how other third parties behave toward them and their tendency to reciprocate these others in subsequent interactions.

Despite these strengths, we need to acknowledge several limitations in our experiment. First, having participants come to the lab at the same time was a bit challenging due to coordination problems. We eventually recruited 240 participants who were divided into 80 triads (Person A, B, and C). Thus, we could examine Person A’s behaviors in two sequential games, Person B’s emotions and evaluations in response to Person A’s behavior, and Person C’s trust decisions toward Person A in the subsequent interaction. While this design enables us to test the behavioral dynamics in sequential dyadic interactions, it lowers our valid sample size and statistical power to test our hypotheses, and may potentially cause an endogeneity issue (e.g., reciprocity in the TG may depend on the trust decisions). To alleviate the endogeneity issue, we included several covariates (e.g., participants’ social value orientation, the trustor’s trust decisions) in our analyses on the behavioral measures. Thus, we can conclude that our experimental manipulations affect participants’ behaviors beyond their own social value orientation or others’ decisions. Of course, future research needs to replicate our findings with larger sample size or use strategy method assuming that participants act as one of the separate roles. Second, we used a trust game to measure subsequent cooperation, which may make behaviors in the two interactions incomparable. While using a dictator game (DG) as the second game may be a better option to test our hypotheses, we believe that switching to the trust game (TG) has its own strengths. For example, the trust game involves sequential decisions made by the trustor and the trustee, and provides more information about the behavioral dynamics among interacting partners, particularly how other third parties (e.g., trustor) behave toward an individual (e.g., trustee) when this individual has experienced a strategic interaction (e.g., UG) or when there is potential gossip about this individual, and the extent to which this individual would reciprocate those others. In addition, behavioral trust and reciprocity in trust games are highly correlated with cooperation in dictator games (Peysakhovich et al., 2014), which validates our use of trust games to test our hypotheses. Third, we used asymmetric games in which two interacting partners made different decisions sequentially, and thus behaviors in these games may not resemble those in symmetric games where people in dyads (e.g., two-person prisoner’s dilemma game) or groups (*n* > 2; e.g., public goods game; Larrick and Blount, 1997) have equal power and make simultaneous decisions. Thus, we need to be cautious when comparing our findings with previous findings observed in symmetric games (e.g., the public goods game; Wu et al., 2016a). Future research may test whether the observed effects in our experiment would also apply in symmetric games involving groups of people by directly comparing asymmetric and symmetric decision contexts.

## Concluding Remarks

Cooperation is essential for groups and organizations to thrive. Our research goes beyond the existing literature on gossip and cooperation by focusing on the dynamics of cooperation in two sequential dyadic interactions, while taking into account gossip and strategic motivations during one’s decisions. Findings suggest that potential gossip by one’s partner significantly increases the amount of resources allocated to the partner, but potential partner rejection does not significantly enhance the observed level of cooperation. In addition, those who have experienced strategic interactions due to fear of rejection by their partners are less likely to cooperate by reciprocating others in subsequent interactions, particularly when they could build a good reputation through their previous behavior. These findings imply that gossip can promote cooperation across strategic and non-strategic situations, but the potential rejection by one’s partner has a weak effect in promoting cooperation, and may undermine future cooperation especially when paired with their partners’ reputation sharing opportunities. Thus, it is important for practitioners and policy-makers to take into account the specific situations that people may encounter when implementing reputation systems with the aim to promote and maintain cooperation in a cost-effective manner. In particular, reputation systems may be a stronger candidate to promote cooperation and to cultivate intrinsic cooperators when people can clearly “communicate” their prosociality to their partner in dyadic interactions.

## Data Availability

The supplementary materials, dataset, and syntax for this research can be found in the Open Science Framework: https://osf.io/ft8mu/.

## Ethics Statement

This experiment was carried out in accordance with APA ethical standards. Participants provided their informed consent prior to taking part in the experiment, and had the opportunity to withdraw at any time during the experiment. The protocol was approved by the Ethics Committee of Vrije Universiteit Amsterdam.

## Author Contributions

JW, DB, and PV had the initial idea for the experiment and designed the experiment. JW collected the data, conducted the data analyses, and wrote the first draft of the paper with the generous input by DB, YK, and PV.

### Conflict of Interest Statement

The authors declare that the research was conducted in the absence of any commercial or financial relationships that could be construed as a potential conflict of interest.

## Footnotes

^1^One male participant returned 10 MUs in the trust game when the trustor sent him 0 MU, which was impossible. Thus, we excluded this participant when analyzing reciprocity.
